# PW01-007 – Colchicine brand switching in FMF patients

**DOI:** 10.1186/1546-0096-11-S1-A60

**Published:** 2013-11-08

**Authors:** J Jagger, J McGonagle

**Affiliations:** 1 Medicine, University of Virginia, Charlottesville, Virginia, USA; 2 Pediatrics, Crotched Mountain Rehabilitation Center, Greenfield, New Hampshire, USA

## Introduction

In July of 2009 the U.S. Food and Drug Administration enacted new regulation of colchicine under the “Unapproved Drugs Program.” Like other old drugs that were on the market before the existence of the FDA, colchicine had never been subjected to FDA-required safety and efficacy trials. One company elected to put colchicine through the FDA’s approval protocol and when approval was granted in 2009 the FDA announced a ban on five unapproved brands of colchicine on the market and gave proprietary rights to one approved brand. The sudden brand changes that followed this regulation led to a therapeutic crisis for FMF patients in the U.S. and coincidentally revealed heretofore unrecognized patterns in patient response to different brands of colchicine.

## Objectives

To document and characterize the brand specific response of individual FMF patients participating in FMF support groups on the internet and determine the potential of multiple brand exposure for improving the effectiveness of colchicine by reducing intolerance and resistance rates.

## Methods

An online survey of FMF patients participating in patient support groups on Facebook and Yahoo was conducted. Patients residing in the U.S. were selected and were asked to report each brand of colchicine they had ever taken and to indicate for each brand whether their response was satisfactory; unsatisfactory, but kept taking it; or unsatisfactory and discontinued it.

## Results

40 FMF patients reported taking 11 brands of colchicine; a total of 101 individual/brand trials. Of 31 patients taking 2 or more brands, 3 brands was the average number tried. FMF patients revealed a highly idiosyncratic response to different brands of colchicine. Adverse responses and ineffectiveness were common for all brands ranging from 81% (FDA approved brand) to 30% (FDA banned brand). A critical finding was that all 9 patients discontinuing a brand for ineffectiveness (“colchicine resistance”) had a satisfactory response to another brand. Despite the frequency of adverse responses (intolerance or ineffectiveness) to individual brands, all patients with exposure to 2 or more brands reported a satisfactory response to at least one brand.

## Conclusion

Intolerance and ineffectiveness (i.e. “colchicine resistance”) are not characteristics of FMF patients but are idiosyncratic brand responses. Most FMF patients could achieve a satisfactory therapeutic response to colchicine if a minimum of three independent brands were available to them. Wider availability of colchicine brand options are needed for all FMF patients. Before resorting to alternative higher-risk drugs, FMF patients should be shown to be intolerant to 3 independent brands of colchicine. Although this is a clinically simple therapeutic strategy, the greater challenge is the maze of regulations governing the importation of pharmaceuticals across national borders.

## Disclosure of interest

None declared

